# Crushed Confluence with Significant Tissue Loss: A Case Report of a Rare Biliary Tract Injury following Blunt Trauma Abdomen

**DOI:** 10.7759/cureus.5235

**Published:** 2019-07-25

**Authors:** Humaid Ahmad, Omema Saleem, Rasheeqa Mahmood, Jahanzaib Haider, Shams Nadeem Alam

**Affiliations:** 1 Surgery, Dow University of Health Sciences, Karachi, PAK; 2 Surgery, Jinnah Medical College Hospital, Karachi, PAK

**Keywords:** traumatic biliary tract injuries, biliary confluence injuries, biliary-enteric anastomoses, grade iv liver injury, polytrauma, polytrauma, road traffic accident

## Abstract

Traumatic extrahepatic biliary tract injuries are rare occurrences because they are a relatively small target of injury following trauma. They are almost always associated with injuries to surrounding structures, which take precedence during initial management. The management of extrahepatic biliary tract injuries depends on the extent and level of the injury. This may include primary repair to high-level biliary-enteric anastomoses. We report a case of injury to the biliary confluence that occurred after blunt trauma abdomen. The injury was associated with an extensive liver injury that was the focus of initial treatment. We discuss the possible mechanisms that led to injury of this relatively hidden area and describe the various treatment options that can be applicable in such cases.

## Introduction

Extrahepatic biliary tract injuries are usually iatrogenic [1], but have been rarely reported after trauma [2]. They can occur after both blunt and penetrating trauma [1]. Traumatic injuries of the extrahepatic biliary tract are commonly associated with injuries to surrounding structures including liver, great vessels, duodenum, and pancreas. While the surrounding injuries take precedence in the initial management of the trauma patients, they do lead to an early diagnosis of the extrahepatic biliary tract injuries as most patients undergo early exploratory laparotomy because of the associated injuries [2]. This early diagnosis, in turn, leads to a better prognosis as compared to the worse prognosis of delayed diagnosis that occurs in patients where conservative management was initially undertaken for blunt trauma. This latter group presents later on with biliary peritonitis/sepsis due to bile leak or obstructive jaundice/ascending cholangitis due to biliary strictures [3]. We report a case of a young male with injury to the biliary confluence located at the upper part of the extrahepatic biliary tract that occurred following blunt trauma abdomen. Fortunately, the diagnosis was made at a relatively early stage in his management.

## Case presentation

**Figure 1:**
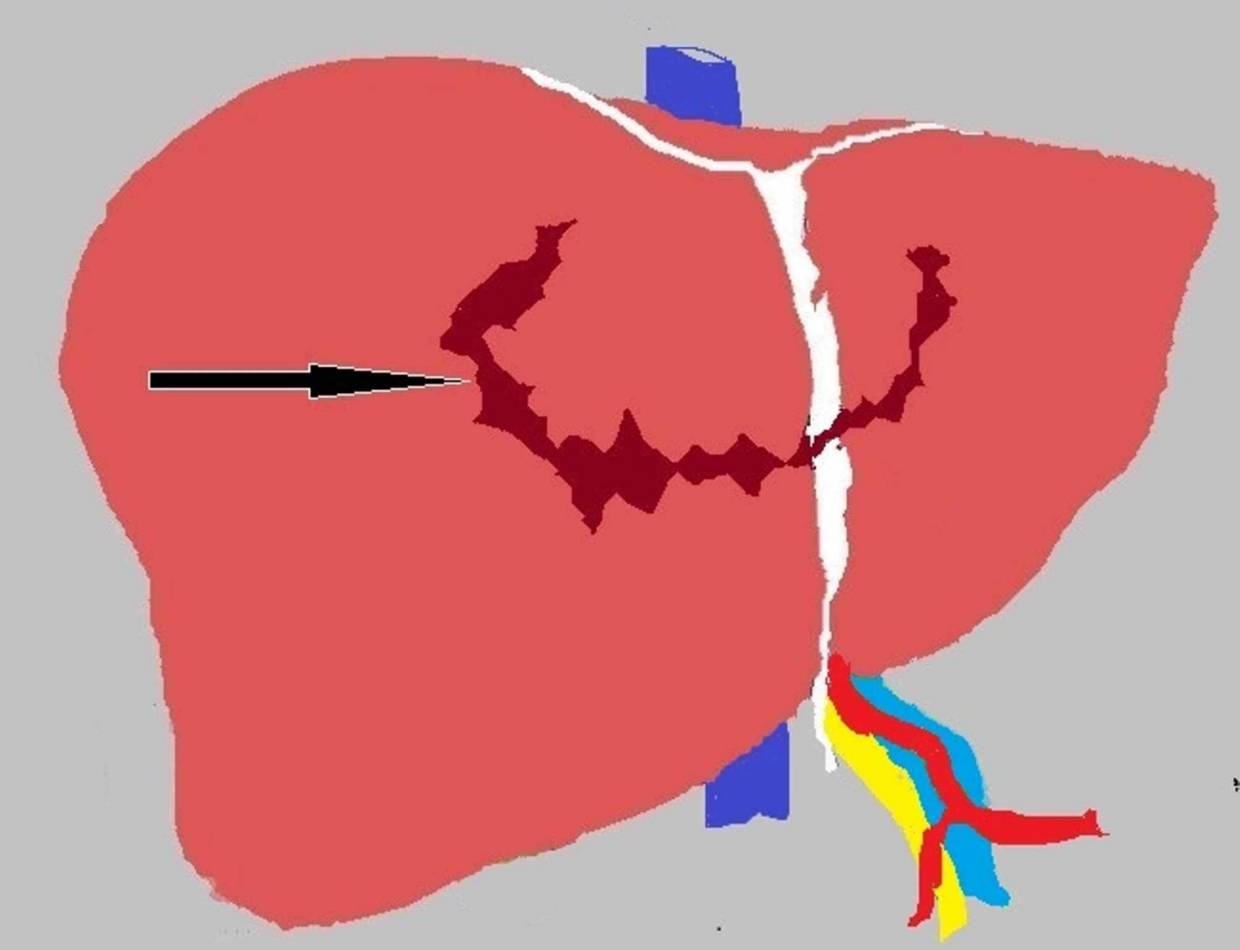
Illustration showing the liver injury encountered upon entering the abdomen during exploratory laparotomy Black arrow shows deep circumferential laceration on the superior surface of the liver crossing the midline to involve both the right and left lobes.

A 28-year-old male was travelling on a motorcycle with his wife. He was involved in a road traffic accident where a truck ran over them. The front bumper of the truck hit his lower chest and upper abdomen and the couple were dragged underneath it. The patient was brought to the emergency in severe respiratory distress and class IV hypovolemic shock. His left forearm was mangled as it had been crushed underneath the front wheel of the truck. After initial resuscitation following the ATLS guidelines including a chest tube intubation for right-sided hemopneumothorax, the patient was urgently shifted to the operation theatre. He underwent exploratory laparotomy by our team while amputation of his left forearm was simultaneously done by orthopedic surgery team. During exploratory laparotomy, three liters of blood was evacuated from the cavity. A liver injury that was found is shown in Figure 1 as an illustration because pictures of the injury were not taken at the time of surgery. This liver injury was the only source of massive blood loss encountered during the surgery.

As the patient remained hemodynamically unstable during the surgery despite multiple blood transfusions and there was ongoing bleeding from the liver, packing of the liver was done and the abdomen was closed to revisit after 48 hours. After a period of 48 hours’ optimization in the surgical ICU, the patient was shifted back to the operation theatre where re-exploration was done electively. On re-exploration, approximately 1800 ml of blood-tinged bile was found in the peritoneal cavity. On removing the packs around the liver, the injury of the liver was re-examined more carefully as now bleeding had significantly reduced and the patient was hemodynamically stable.

**Figure 2:**
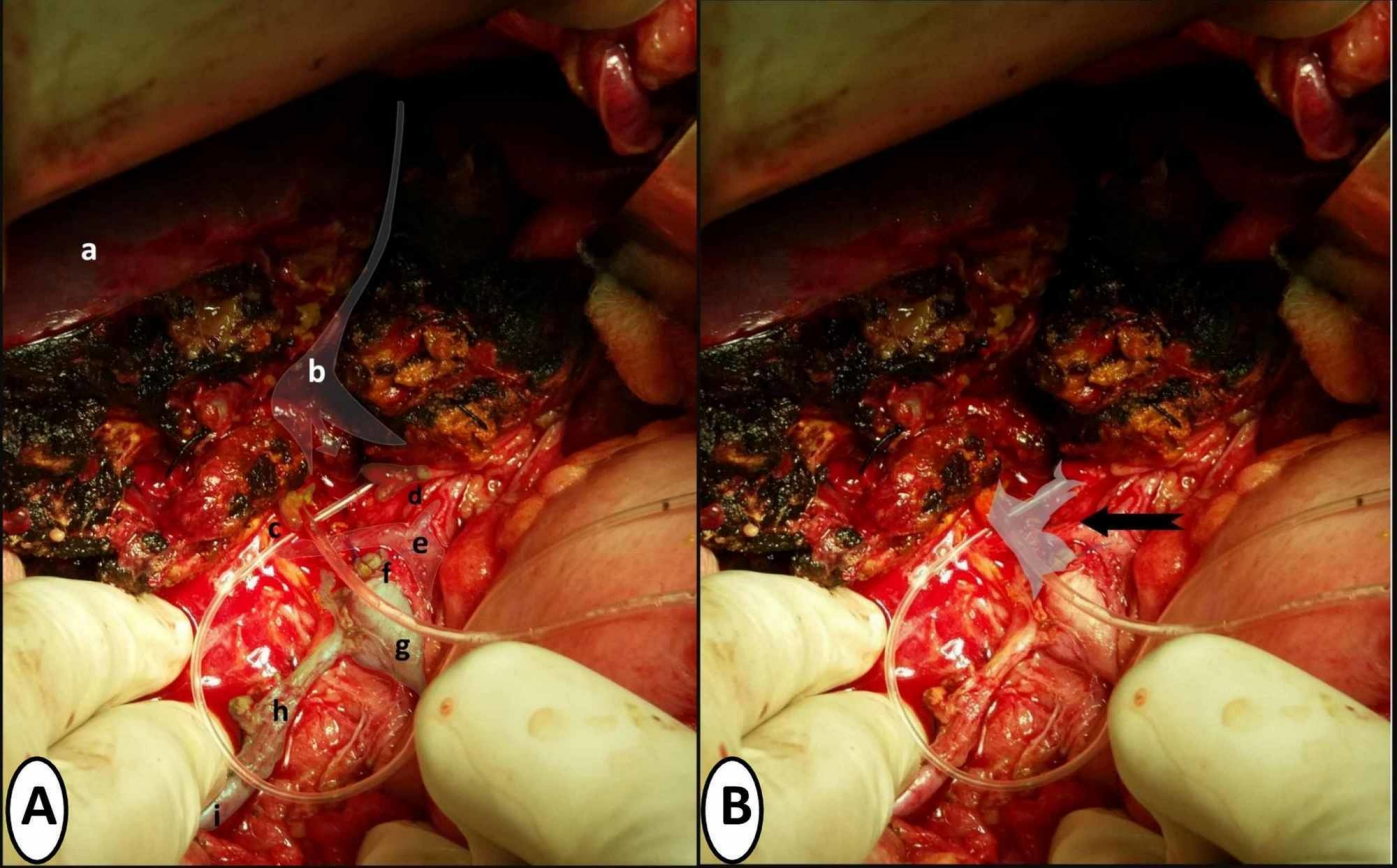
Per-operative pictures showing related anatomical structures and loss of biliary confluence post-debridement of ischemic necrotic tissue Figure 2A highlights related anatomical structures labeled as follows: a shows the superior surface of the right lobe of the liver. b shows the line of division of liver parenchyma that started from the liver surface down to the liver hilum. c shows proximal right hepatic duct post-debridement with the feeding tube placed in its lumen. d shows proximal left hepatic duct post-debridement with the feeding tube placed in its lumen. e shows the common hepatic artery with bifurcation into right and left hepatic arteries. f shows the ligated upper end of common hepatic duct post-debridement. g shows the common bile duct. h shows the cystic duct. i shows the gallbladder. Figure 2B shows the area of significant tissue loss of the biliary tract marked by the black arrow, which includes the distal parts of the left and right hepatic ducts, biliary confluence and proximal part of the common hepatic duct.

Upon examination of the liver, bile mixed with a small amount of blood was seen coming out of the liver laceration. A hematoma was also found at the liver hilum, which was partially walled off by the adjacent duodenum and stomach. It was decided to explore the liver laceration to secure hemostasis as well as ligate any leaking bile ducts on both sides of the laceration. The liver injury was a grade IV laceration as it was more than 10 cm deep. Slowly and gradually as dissection proceeded deeper into the laceration, small bleeding vessels of the liver parenchyma were ligated. However, no major source of bile leak was identified. Bile seems to be coming from the depth of the liver laceration. In view of this, it was decided to divide the liver from the laceration downwards at the Cantlie's line so that the liver could be split into anatomical right and left lobes. Dissection was continued down until a damaged Glisson’s capsule was encountered with the right and left hepatic ducts identified converging on an area of very little unhealthy and ischemic tissue. This unhealthy tissue was attached to the common bile duct further down and was identified as remains of the confluence of the biliary tract. Debridement was done to the edges of the healthy left and right hepatic ducts above and the common hepatic duct below. The upper end of the common hepatic duct was closed. Figure 2A shows the line of division of parenchyma and anatomical structures encountered at the liver hilum. Figure 2B is the similar picture but shows the area of the biliary tract that was probably lost due to the injury including distal parts of the left and right hepatic ducts, the biliary confluence, and the proximal part of the common hepatic duct. The patient then underwent a Roux-en-Y double barrel (separate left and right cholangiojejunostomy) biliary-enteric anastomosis as the greater distance between the right and left hepatic ducts did not allow the formation of a neo-confluence.

After all hemostasis was secured and formal exploratory laparotomy was completed, the abdomen was closed and the patient was shifted back to Surgical ICU. The patient tolerated the procedure well. He had a difficult postoperative course and prolonged hospital stay due to bilateral lung contusions and sepsis, but eventually recovered. His right hypochondrial drain showed less than 50 ml bile-tinged fluid on the first postoperative day, which gradually settled till 10th postoperative day. His stay in the ICU was for 14 days and he was discharged from the ward on the 31st postoperative day of re-exploration.

At one-year follow-up, the patient has not developed any complications of the biliary-enteric anastomoses.

## Discussion

Extrahepatic biliary tract injuries due to blunt trauma abdomen are rare [1]. They have a reported incidence of 2% to 5% after trauma to the abdomen. They are usually associated with injuries to adjacent organs. The exact mechanism of injury is unknown; however, various causes have been hypothesized to influence the process [2]. These include increased intra-ductal pressure due to compression of the gallbladder with the resultant ductal blowout, biliary tract compression against the spine due to the crushing force, and shearing of biliary tract from cephalic and caudal displacement as a result of rapid deceleration [1,3-4]. In this patient, the authors feel the following series of events occurred that led to this rare injury. The initial impact to the patient’s upper abdomen and lower chest may have caused compression of the confluence of the biliary tract against the patient’s spine by force transmitted through the liver. This is evidenced by the deep circumferential laceration on the superior surface of the liver across the midline that was caused by sudden inward movement of the lower ribcage and xyphisternum against the liver. Deep exploration and dissection through this laceration during the second surgery also led to the liver hilum. This in turn probably led to the crushing of the confluence without bile leak rather than the avulsion mechanism because bile was not found in the peritoneal cavity or the supracolic compartment at the time of initial exploration. As time passed, the damaged confluence became necrotic due to ischemia related to thrombosis of damaged vasculature around the hilum. The necrotic area then sloughed off in the ensuing hours, causing bile to accumulate in the peritoneal cavity that was discovered on re-exploration 48 hours later. A simultaneous inflammatory reaction also walled off the hilum of the liver during the first 48 hours, causing bile to track into the peritoneal cavity through the only way that was possible, that is, through the laceration of the liver. It is unfortunate that the authors did not take a picture of the liver injury initially found during re-exploration, which is now shown as an illustration (Figure 1) so that the injury can be appreciated by readers. However, the authors did not expect the resultant biliary tract injury to be so severe. It was thought that bile was leaking from transected small biliary channels in the circumferential parenchymal laceration of the liver.

Minor injuries of the biliary tract can be managed with a primary repair or end-to-end anastomosis [2]. However, with regard to biliary tract injuries having significant tissue loss, continuity of the tract requires Roux-en-Y biliary-enteric anastomoses as long as the patient is hemodynamically stable. In hemodynamically unstable patients, the goal should be external drainage with a plan to re-explore when the patient is more optimized [5]. Consequently in our patient, even if this biliary tract injury had been evident during the initial exploration, we would have opted with drainage and repair later, rather than initial repair. With regard to creating a high-quality biliary-enteric anastomosis, the same principles may be applied that are essential in cases of anastomosis for iatrogenic injury of the confluence. This includes debridement of dead and ischemic tissue to healthy bleeding edges of the biliary tract [6]. The resultant defect in the biliary tract dictates the choice between the creation of a neo-confluence, portoenterostomy or formation of separate left and right hepatic duct anastomosis also called double-barrel anastomosis. The decision is based on the amount of tension created in the anastomosis which should be as little as possible to avoid anastomotic breakdown later on [6]. In our case, after debridement to healthy biliary tract walls, the distance between the left and right ducts was too much to allow a tension-free anastomosis between them (Figure 2B). Therefore, it was decided to undertake double-barrel anastomosis to ensure tension-free repair rather than creating neo-confluence between left and right hepatic ducts that would have tension. Portoenterostomy was not considered in our case as there was enough length of right and left hepatic ducts to allow safe and secure anastomosis. It is an option deemed necessary when more than 50% of the anastomosis cannot be done without complete apposition epithelium and mucosa of bile ducts and small bowel [6].

It is worth mentioning here that the main vessels of the liver remained spared from injury in our patient (Figure 2A). While concomitant injury to these vessels would mean a fatal outcome for such patients, it has been noted in the past that they tend to remain spared after blunt trauma. It is postulated that this is related to the relative elasticity of hepatic arteries and extra length of portal veins in comparison to the biliary tract [5]. However, the fact that a substantial length of left hepatic, right hepatic and common hepatic duct required debridement shows that vasculature in the area of the confluence was significantly damaged. This damaged vasculature consists of the communicating arcade between the left and right hepatic arterial system, is located within the hilar plate around the confluence, and plays an essential role in the blood supply of the confluence [7-8].

Patients with biliary tract injuries can face a number of sequelae following the restoration of continuity of the tract by any method. These include anastomotic leak and infection in the short term to biliary strictures and cholangitis in the long term. In the event of these complications, re-attempt at anastomosis can pose a significant surgical difficulty if the complications are not managed conservatively [1,9]. Such issues can be avoided if surgeons trained in hepatobiliary surgery are involved early on in these cases [1, 6]. While there was a greater risk of development of post-anastomotic strictures in the option we chose [6], our patient has remained symptom free on one-year follow-up.

## Conclusions

Extrahepatic biliary tract injuries after blunt trauma abdomen are rare. Crushed confluence can occur as a result of severe compression of lower ribcage against the spine and should be suspected if a large circumferential laceration of the superior surface of the liver is discovered intraoperatively. Injury to main biliary tract can be missed on initial exploration especially if tract is crushed as bile leak may not be evident until after injured and ischemic areas of the tract become necrotic and slough off. Repair in such cases requires restoration of biliary tract continuity with a biliary-enteric anastomosis if there is significant tissue loss. Repair done by surgeons trained in hepatopancreatobiliary surgery can produce better postoperative outcome.
